# Optimization on machine learning based approaches for sentiment analysis on HPV vaccines related tweets

**DOI:** 10.1186/s13326-017-0120-6

**Published:** 2017-03-03

**Authors:** Jingcheng Du, Jun Xu, Hsingyi Song, Xiangyu Liu, Cui Tao

**Affiliations:** 1 The University of Texas School of Biomedical Informatics, 7000 Fannin St Suite 600, Houston, TX 77030 USA; 2 The University of Texas School of Public Health, 1200 Pressler Street, Houston, TX 77030 USA

**Keywords:** Twitter, Social media, Sentiment analysis, Support vector machines, Hierarchical classification, Gold standard

## Abstract

**Background:**

Analysing public opinions on HPV vaccines on social media using machine learning based approaches will help us understand the reasons behind the low vaccine coverage and come up with corresponding strategies to improve vaccine uptake.

**Objective:**

To propose a machine learning system that is able to extract comprehensive public sentiment on HPV vaccines on Twitter with satisfying performance.

**Method:**

We collected and manually annotated 6,000 HPV vaccines related tweets as a gold standard. SVM model was chosen and a hierarchical classification method was proposed and evaluated. Additional feature sets evaluation and model parameters optimization was done to maximize the machine learning model performance.

**Results:**

A hierarchical classification scheme that contains 10 categories was built to access public opinions toward HPV vaccines comprehensively. A 6,000 annotated tweets gold corpus with Kappa annotation agreement at 0.851 was created and made public available. The hierarchical classification model with optimized feature sets and model parameters has increased the micro-averaging and macro-averaging F score from 0.6732 and 0.3967 to 0.7442 and 0.5883 respectively, compared with baseline model.

**Conclusions:**

Our work provides a systematical way to improve the machine learning model performance on the highly unbalanced HPV vaccines related tweets corpus. Our system can be further applied on a large tweets corpus to extract large-scale public opinion towards HPV vaccines.

**Electronic supplementary material:**

The online version of this article (doi:10.1186/s13326-017-0120-6) contains supplementary material, which is available to authorized users.

## Background

Human papillomavirus (HPV) is thought to be responsible for more than 90% of anal and cervical cancers, 70% of vaginal and vulvar cancers, and more than 60% of penile cancers [1]. FDA approved HPV vaccines (Gardasil, Cervarix and Gardasil 9) for the protection from most of the cancers caused by HPV infections. However, the HPV vaccines coverage in USA is still quite low especially for the adolescents. Only 39.7% of girls and 21.6% of boys have received all three required doses [2]. Analysis of public opinions over the HPV vaccines could reveal the reasons behind the low coverage rate and can help us provide new directions on improving future HPV vaccines uptake and adherence.

As one of the most popular social media in the world, Twitter attracts millions of users to share opinions on various topics every day. On average, around 6,000 tweets are tweeted every second and 500 million tweets are tweeted per day [3]. Besides, Twitter allows a limit of 140 characters on one post to its users. This restriction pushes the users to be very concise to share their opinions [4]. The huge number of concise tweets makes Twitter a precious and rich data source to analyze public opinions [5].

Due to the adaptability and accuracy, machine learning based approach is one of the most prominent techniques gaining interest in sentiment analysis (SA) on microblogging posts [4]. However, few efforts have been done on Twitter to explore public opinions towards vaccines using machine learning based SA tools. Surian et al. applied unsupervised topic modeling to group semantically similar topics and communities from HPV vaccines related tweets [6]. However, those topics are not closely related to sentiments towards vaccination. Salathé et al. leveraged several supervised algorithms to mine public sentiments toward the new vaccines [7]. Zhou and Dunn et al. utilized connection information on social network to improve opinion mining on identifying negative sentiment about HPV vaccines [8, 9]. However, those work only covered limited coarse sentiment classifications (positive, negative, neutral, etc.). In the HPV vaccination domain, sentiment analysis at a more granular level is necessary in addition to the current limited classifications. To serve as a feedback to public health professionals to examine and adjust their HPV vaccines promotion strategies, the system not only needs to know whether people have negative opinions towards HPV vaccines but also should be able to extract the reasons behind the negative opinions.

Thus, to access public opinions towards HPV vaccines on Twitter in a more comprehensive way, a finer classification scheme to HPV vaccination sentiment is needed. In this paper, we introduced our efforts on using machine learning algorithms to access HPV vaccination sentiment at a more granular level on Twitter. We built a hierarchical classification scheme including 10 categories. To train the machine learning model, we manually annotated 6,000 tweets as the gold standard according to the classification scheme. We chose Support Vector Machines (SVM) as the algorithm due to the performance in our pre-experiments. Due to the challenges of machine learning approaches on the highly unbalanced tweets corpus, we further did a series of optimization steps to maximize the system performance. Standard metrics including precision, recall, and F measure were calculated to evaluate our results.

## Methods

### Data source and annotation

#### Data collection

English tweets containing HPV vaccines related keywords were collected from July 15, 2015 to August 17, 2015. We used combinations of keywords (HPV, human papillomavirus, Gardasil, and Cervarix) to collect public tweets using the official Twitter application programming interface (API) [10]. During the study period, we have collected 33,228 tweets in total. After removing the URLs and duplicate tweets, we randomly selected 6,000 tweets for annotation.

#### Annotation schema design

**Fig. 1:**
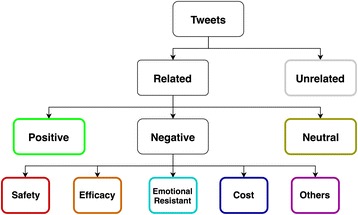
Sentiment classification scheme for HPV vaccines related tweets: The categories in colored rectangles (other than black) are all possible sentiment labels that can be assigned to the tweets

As we’re more interested in the concerns over HPV vaccination, we did a literature review to find out the common non-vaccination reasons of HPV vaccines [11–14]. The most common barriers found for vaccination are the worries about side effects, efficacy, cost, and culture-related issues. We also went through a sample of tweets and kept track of the major concerns on Twitter. Based on our findings, a hierarchical classification scheme was then built for the classifications of different HPV vaccination sentiments, see Fig. 1. Detailed definitions of each category were provided in Table 1.

**Table 1:** Detailed definition of different sentiment categories for HPV vaccines related tweets

Sentiment		Description
Positive		Show positive opinion or prompt the uptake of HPV vaccine
Negative	Safety	Concerns or doubt on the safety issues of HPV vaccine or present vaccine injuries
	Efficacy	Concerns or doubt on the effectiveness of HPV vaccine
	Cost	Concerns on the cost of HPV vaccine (e.g.: money or time)
	Resistant	Resistance to HPV vaccines due to cultural or emotional issues
	Others	Other concerns
Neutral		Related to HPV vaccine topic but contains no sentiment or sentiment is unclear or contains both negative and positive sentiment
Unrelated		Not related to HPV vaccine topic

#### Gold standard annotation

We annotated each tweet based on its content. Three annotators (part time) were employed in this annotation process. Two of them have a public health background and the other has health informatics background. The annotators annotate the tweets according to the classification scheme. The annotator first decides whether the tweet is related to HPV vaccines or not. If it is related, the annotator further decides if it is positive, negative, or neutral. If it is negative, the annotator assigns one of the categories under “Negative” to the tweet.

All tweets have been annotated by at least two annotators in the first round. The third annotator was involved when the two annotators have different annotations and made the final decision in the second round. The first round took up to one month. The second round took up to two weeks. We applied the brat rapid annotation tool for this process [15]. After the annotation, the Kappa value was calculated from the annotators to evaluate the quality [16].

The example tweets annotated in our gold standard can be seen in the Additional file 1: Table S1A.

### Machine learning system optimization

**Fig. 2:**
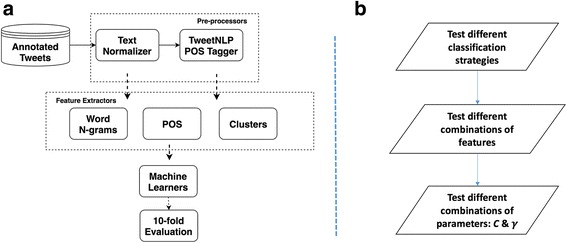
Overview of the machine learning based system and optimization approach: (**a**) modularized machine learning system framework; (**b**) machine learning optimization steps

Our system is a modularized machine learning system that consists different pre-processors and feature extractors. A detailed overview of the system can be seen in Fig. 2a.

#### Tweets Pre-processing

- Text Normalizer. All upper-case letters were converted to lower case ones. All hashtags and Twitter user names (e.g. @twitter) were excluded. All URLs were exchanged with string “url” (e.g. ‘http://example.com’ to ‘url’). We also replaced any letter occurring more than two times in a row with two occurrences (e.g. convert ‘huungry’, ‘huuuungry’ to ‘huungry’), proposed by Go A et al. [17].
- POS Tagger. We used TweeboParser [18, 19] developed by Carnegie Mellon University to extract POS tags for tweets. TweeboParser is trained on a subset of new labeled corpus for 929 tweets (12,318 tokens) [19]. It provided a fast and robust Java-based tokenizer and POS tagger for tweets.

#### Features extraction

Considering the characteristics of HPV vaccine related tweets, we extracted the following features:

- Word n-grams. Contiguous 1 and 2 g of words are extracted from a given tweet.
- Clusters. Previous work found that word cluster can be used to improve the performance of supervised NLP models [20]. We mapped tweets tokens to TwitterWord Clusters developed by ARK group of Carnegie Mellon University (the group is currently in University of Washington). This largest clustering mapped 847,372,038 tokens from approximately 56 million tweets into 1000 clusters. (e.g. “tehy", “thry”, “theey”, “they” et al. belong to a same cluster)
- POS tags. Part of speech tags were extracted by TweeboParser as one of the features.

#### Machine learning algorithm

In our pre-experiment, we leveraged the basic n-grams feature and applied Weka [21] to test and compare different machine learning algorithms: Naïve Bayes, Random Forest and Support Vector Machines (SVMs). As SVMs outperformed the other two algorithms and it has known performance on pervious sentiment analysis tasks [22], we leveraged SVMs as the algorithms. SVMs are supervised learning models with associated learning algorithms that analyze data used for classification and regression analysis. We implemented LibSVM package as the library for our task. Default RBF kernel was used.

#### Machine learning system optimization

- Baseline model. To create a baseline sentiment analysis model, we applied plain classification, used word-ngrams as the feature and chose default SVMs parameters.
- Hierarchical classification VS plain classification. Traditional multi-labels classification methods that treat each category equally do not take into account the hierarchical information. The highly imbalanced structure of our gold standard could have a dramatic effect on the system performance [18]. In order to alleviate the effect of the imbalanced structure, we tested the hierarchical classification and compared the performance with the plain one. Three SVMs models were trained independently. The first SVM model categorized the tweets into “Related” and “Unrelated” groups; the second one then categorized the “Related” tweets into “Positive”, “Negative” and “Neutral” groups; the third model further categorized the “Negative” tweets into the five finest categories.
- Feature combinations. We tested the different combinations of word n-grams, clusters and POS tags features and evaluated their impact on the system performance.
- Parameters optimization. For SVMs model with RBF kernel, there are two major parameters needed to be chosen beforehand for a given problem: *C* is the cost of misclassification; $\boldsymbol{\gamma}$ is the parameter of the kernel function [19]. The *C* parameter trades off misclassification of training examples against simplicity of the decision surface, while the $\boldsymbol{\gamma}$ defines how far the influence of a single training example reaches, with low values meaning ‘far’ and high values meaning ‘close’ [23].

An overview of the optimization steps can be seen in Fig. 2b.

#### Evaluation

To evaluate the performance of the machine learning algorithms, we used 10-fold cross-validation. Standard metrics were applied and the average score were calculated (including precision, recall and F measure for each category and Micro F measure and Macro F measure for overall performance). For micro-averaged score, we summed up all the individual true positives, false positives, and false negatives of the system. For macro-averaged score, we took the average of the F score of different classes.

## Results

### Annotation results

**Fig. 3:**
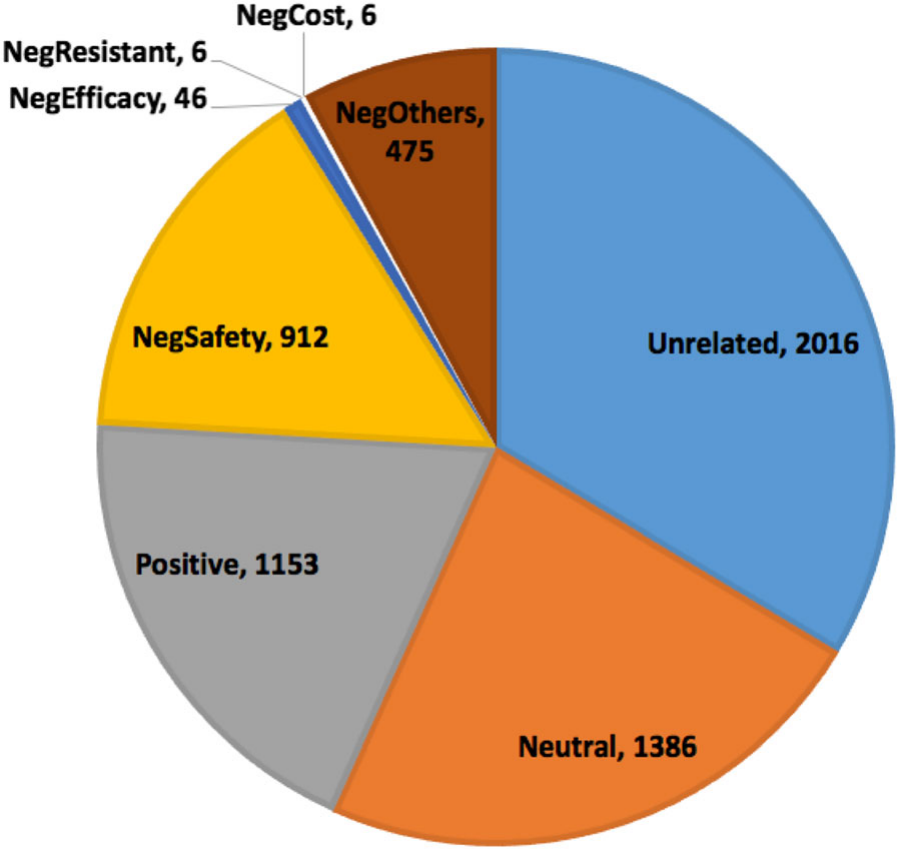
Sentiment distribution in 6,000 tweets gold standard. (Neg: Negative)

The Kappa value among the annotators was 0.851, which indicated the high quality of this gold standard. Among the human annotated corpus, 3,984 (66.4%) tweets were related to HPV vaccine sentiments. Among the related tweets, 1,445 (36.3%) of them showed negative opinions, which is larger than both positive (1,153, 28.9%) and neutral tweets (1,386, 34.8%). The major concern in gold standard is safety issues (63.1% in Negative group). Detailed results can be seen in Fig. 3. The download link for annotation results can be found in section “Availability of data and material”.

### Machine learning system optimization results

#### Baseline model performance

Choosing word-ngrams as the feature and default SVMs parameters (C = 256 and $\boldsymbol{\gamma}$ = 2e-5), we applied the traditional plain classification to create the baseline model.

#### “Hierarchical” VS “Plain”

The performance comparison between baseline model (plain classification) and hierarchical classification can be seen in Table 2. The hierarchical classification method outperformed the plain method in each category. For the micro-averaging and macro averaging F score, hierarchical way significantly increased the performance to 0.7208 and 0.4841 from 0.6732 and 0.3967 respectively. Specifically, for the category “NegOthers” and “NegEfficacy”, the hierarchical method increased 0.3095 and 0.2593 on F score respectively.

**Table 2:** 10-fold cross validation performance on the baseline model and hierarchical classification model. (F: F-1 score; P: precision; R: recall; for the categories that do not indicate the metric, F-1 score are used)

Classification Model		Plain Classification (Baseline model)	Hierarchical Classification
Micro-averaging	F	0.6732	0.7208
Macro-averaging	P	0.4455	0.5402
	R	0.3574	0.4386
	F	0.3967	0.4841
Unrelated		0.8044	0.8599
Neutral		0.5792	0.6181
Positive		0.6528	0.7021
NegSafety		0.7006	0.7277
NegEfficacy		0	0.2593
NegCost		0	0
NegResistant		0	0
NegOthers		0.155	0.4645

#### Results for the evaluation on feature sets

Since the hierarchical method outperformed the plain method significantly, we chose this way as default in our following optimization steps. Default SVMs parameters (*C* = 256 and $\boldsymbol{\gamma}$ = 2e-5) were used in this step. The 10-fold evaluation results for different feature sets combinations can be seen in Table 3.

**Table 3:** 10-fold cross validation performance on different feature sets combinations. (Feature sets: (a) Word n-grams; (b) POS tags; (c) Clusters; F: F-1 score; P: precision; R: recall; for the categories that do not indicate the metric, F-1 score are used)

Feature sets		(a)	(a) + (b)	(a) + (c)	(a) + (b) + (c)
Micro-averaging	F	0.7208	0.7263	0.7255	0.73
Macro-averaging	P	0.5402	0.5438	0.5396	0.5477
	R	0.4386	0.4468	0.4442	0.4576
	F	0.4841	0.4905	0.4872	0.4986
Unrelated		0.8599	0.864	0.859	0.8618
Neutral		0.6181	0.6226	0.625	0.6231
Positive		0.7021	0.7098	0.7123	0.7136
NegSafety		0.7277	0.734	0.7357	0.7542
NegEfficacy		0.2593	0.3214	0.2593	0.3793
NegCost		0	0	0	0
NegResistant		0	0	0	0
NegOthers		0.4645	0.4614	0.4724	0.4753

The highest micro-averaging and macro-averaging F score were 0.73 and 0.4986, achieved by using the combination of n-grams, POS, and word clusters features. Adding POS and cluster feature set can both lead to nearly 0.5% increase in micro-averaging F -score compared with using word n-grams feature only (POS: from 0.7208 to 0.7263; Cluster: from 0.7208 to 0.7255). Adding POS feature only achieved the highest performance for “Unrelated” category, whereas adding cluster feature outperformed on “Neutral” category. Except for “Unrelated” and “Neutral” category, Adding POS and cluster feature sets together achieved the highest performance.

#### Results for the Evaluation on Parameters Optimization

As adding POS and cluster feature sets together achieved the best performance. The ideal way to find the best parameters *C* and $\boldsymbol{\gamma}$ should be grid search method. However, as we chose the hierarchical classification methods, we need to train three SVMs models independently. The grid search method will be much computation-costly. To reduce the computation burden, we decided to optimize the parameters in two steps: 1) use the default *C* and grid search best $\boldsymbol{\gamma}$ combinations for three SVMs models; 2) use the $\boldsymbol{\gamma}$ combinations that achieved the best performance in step 1 and grid search best *C* combinations for three SVMs models.

The default *C* and $\boldsymbol{\gamma}$ are 256 and 2e-5 respectively. For the step one, we fix *C* to 256 for all the three models and gave $\boldsymbol{\gamma}$ a range of {2e-7, 2e-6, 2e-5, 2e-4, 2e-3} for the grid search. Since we have three models, we totally tested 125 models in this step. The best $\boldsymbol{\gamma}$ combination is: 2e-5 for the first SVMs model, 2e-4 for the second one and 2e-4 for the third one. For the step two, we chose the found $\boldsymbol{\gamma}$ combination in the step one and gave *C* a range of {64, 128, 256, 512, 1024} for the grid search. Due to the three models we have, 125 models were tested in this step. The best *C* combination found is: 512 for the first SVMs model, 128 for the second one and 512 for the third one. The performance comparison between the best performing models after parameter optimization and the model using default parameters can be seen in Table 4. We can observe that by doing parameters optimization, our machine learning model has increased 1.442% and 8.97% on micro-averaging and macro-averaging F score respectively. The optimized model leads to significant increase on nearly all categories except for “NegResistant” category.

**Table 4:** 10-fold cross validation performance among the best performing model after *C* and $\boldsymbol{\gamma}$ optimization and the model using default *C* and $\boldsymbol{\gamma}$. (F: F-1 score; P: precision; R: recall; for the categories that do not indicate the metric, F-1 score are used)

Model		Model using default *C* and $\boldsymbol{\gamma}$	Best model using optimized $\boldsymbol{\gamma}$ only	Best model using optimized *C* and $\boldsymbol{\gamma}$
Micro-averaging	F	0.73	0.7352	0.7442
Macro-averaging	P	0.5477	0.6889	0.6873
	R	0.4576	0.5095	0.5142
	F	0.4986	0.5858	0.5883
Unrelated		0.8044	0.8538	0.8633
Neutral		0.5792	0.6330	0.6470
Positive		0.6528	0.7239	0.7255
NegSafety		0.7006	0.7641	0.7617
NegEfficacy		0	0.4138	0.4068
NegCost		0	0.5	0.5
NegResistant		0	0	0
NegOthers		0.155	0.5144	0.5403

## Discussions

Annotation results showed that there were still many concerns over the HPV vaccine on Twitter during the study period. The number of tweets holding negative opinions on HPV vaccines exceeded the tweets holding positive opinions. The major concern found was about safety issues. As it is a relative small corpus, in the future, we plan to apply this system on a large-scale tweets corpus. We can leverage further analysis tool to track the changes and to identify the patterns of different sentiments toward HPV vaccines over the time.

As the gold standard has a highly imbalanced structure (highly uneven distribution of different categories), traditional plain classification method can’t take advantage of the hierarchical classification information. The proposed hierarchical classification method outperformed the plain method significantly on overall performance and on each category as well. Adding POS tags and word clusters as a feature has already shown its effect on improving performance on previous NLP tasks. Our experiment further demonstrated its power in the multi-classification tasks on tweets corpus for accessing vaccination purpose. Parameter optimization is very necessary according to our results. It can greatly influence the system performance, especially on some categories with very limited number.

There are still several limitations of the work reported here. A serious issue for our Twitter corpus is that it is highly unbalanced, which means that the distribution of different classes is highly diverse. It is very challenging for machine learning system to handle classes with very limited number. In the future, we plan to collect incorporate more tweets of minority classes to the gold standard. In this work, we only used three feature sets. More feature sets can be included to improve the performance, including character n-grams, word dependency, structure feature, and sentiment lexicons feature. Rule-based approaches might be more effective for classification on minority classes. A hybrid system consisting of both machine learning and rule-based approach is supposed to be very helpful.

## Conclusions

We designed and conducted a study to classify HPV vaccine related tweets by the sentiment polarity using machine learning methods. A hierarchical scheme was proposed for different sentiment classifications of HPV vaccines. Ten different categories were included to cover most types of public opinions for HPV vaccines. A gold standard that is consisted of 6,000 randomly selected tweets were manually annotated as the training dataset. Different classification methods were evaluated. Different combinations of feature sets and parameters were tested to optimize the performance of the machine learning model. Compared with the baseline model, the hierarchical classification model with optimized feature sets and model parameters has increased the micro-averaging and macro-averaging F score from 0.6732 and 0.3967 to 0.7442 and 0.5883 respectively.

Our work provides a systematical way to improve the machine learning model performance on the highly unbalanced HPV vaccine related tweets corpus. Our system can be further applied on a large tweets corpus to extract large-scale public opinion towards HPV vaccines. Similar systems can be developed to explore other public health related issues.

## Additional file

Additional file 1:
**Table A.** Sample tweets annotated in the gold standard for each sentiment category (DOCX 43 kb)

## References

[1] Centers for Disease Control and Prevention. HPV-Associated Cancers Statistics [Internet]. Available from: http://www.cdc.gov/cancer/hpv/statistics/index.htm. Accessed July 2016.

[2] Farmar AL, Love-Osborne K, Chichester K, Breslin K, Bronkan K, Hambidge SJ. Achieving High Adolescent HPV Vaccination Coverage. Pediatrics. 2016;5:e20152653. 10.1542/peds.2015-2653 27940751

[3] Twitter Usage Statistics [Internet]. Available from: http://www.internetlivestats.com/twitter-statistics/. Accessed Feb 2017.

[4] Thakkar H, Patel D. Approaches for sentiment analysis on twitter: A state-of-art study. arXiv preprint arXiv:1512.01043. Accessed 3 Dec 2015.

[5] Pak A, Paroubek P. Twitter as a Corpus for Sentiment Analysis and Opinion Mining. InLREc 2010;10(2010).

[6] Surian D, Nguyen DQ, Kennedy G, Johnson M, Coiera E, Dunn AG (2016). Characterizing twitter discussions about HPV vaccines using topic modeling and community detection. J Med Internet Res.

[7] Salathé M, Khandelwal S (2011). Assessing vaccination sentiments with online social media: Implications for infectious disease dynamics and control. PLoS Comput Biol.

[8] Dunn AG, Leask J, Zhou X, Mandl KD, Coiera E. Associations between exposure to and expression of negative opinions about human papillomavirus vaccines on social media: an observational study. J Med Internet Res. 2015;17(6). 10.2196/jmir.4343 PMC4526932 26063290

[9] Zhou X, Coiera E, Tsafnat G, Arachi D, Ong MS, Dunn AG (2015). Using social connection information to improve opinion mining: Identifying negative sentiment about HPV vaccines on Twitter. Stud Health Technol Inform.

[10] API Overview [Internet]. Available from: https://dev.twitter.com/overview/api. Accessed Feb 2017.

[11] Kester LM, Zimet GD, Fortenberry JD, Kahn JA, Shew ML (2013). A national study of HPV vaccination of adolescent girls: rates, predictors, and reasons for non-vaccination. Matern Child Health J Springer.

[12] Zimet GD, Weiss TW, Rosenthal SL, Good MB, Vichnin MD (2010). Reasons for non-vaccination against HPV and future vaccination intentions among 19–26 year-old women. BMC Womens Health.

[13] Holman DM, Benard V, Roland KB, Watson M, Liddon N, Stokley S (2014). Barriers to human papillomavirus vaccination among US adolescents: a systematic review of the literature. JAMA Pediatr.

[14] Why Some Parents Are Refusing HPV Vaccine For Their Children [Internet]. Available from: https://shotofprevention.com/2013/08/20/why-some-parents-are-refusing-hpv-vaccine-for-their-children/. Accessed Aug 2013.

[15] Stenetorp P, Pyysalo S, Topić G, Ohta T, Ananiadou S, Tsujii JI. BRAT: a web-based tool for NLP-assisted text annotation. InProceedings of the Demonstrations at the 13th Conference of the European Chapter of the Association for Computational Linguistics. Association for Computational Linguistics. 2012;23:102-7.

[16] Bhowmick PK, Mitra P, Basu A. An agreement measure for determining inter-annotator reliability of human judgements on affective text. InProceedings of the Workshop on Human Judgements in Computational Linguistics. Association for Computational Linguistics. 2008;23:58-65.

[17] Go A, Bhayani R, Huang L. Twitter sentiment classification using distant supervision. CS224N Project Report, Stanford. 2009;1(12).

[18] Ghazi D, Inkpen D, Szpakowicz S. Hierarchical versus flat classification of emotions in text. InProceedings of the NAACL HLT 2010 workshop on computational approaches to analysis and generation of emotion in text. Association for Computational Linguistics. 2010;5:140-6.

[19] Hsu C-W, Chang C-C, Lin C-J, others. A practical guide to support vector classification. 2003

[20] Turian J, Ratinov L, Bengio Y. Word representations: a simple and general method for semi-supervised learning. InProceedings of the 48th annual meeting of the association for computational linguistics. Association for Computational Linguistics. 2010;11:384-94.

[21] Hall M, Frank E, Holmes G, Pfahringer B, Reutemann P, Witten IH (2009). The WEKA data mining software: an update. ACM SIGKDD Explor.

[22] Xu J, Zhang Y, Wu Y, Wang J, Dong X, Xu H. Citation sentiment analysis in clinical trial papers. InAMIA Annual Symposium Proceedings. American Medical Informatics Association. 2015;2015:1334. PMC4765697 26958274

[23] sklearn.svm. SVR [Internet]. Available from: http://scikitlearn.org/stable/modules/generated/sklearn.svm.SVR.html. February 2017.

