# Supplementary material for: Optimization on machine learning based approaches for sentiment analysis on HPV vaccines related tweets
Source: J Biomed Semantics. 2017 Mar 3;8:9. doi: 10.1186/s13326-017-0120-6 (PMC5335787; doi:10.1186/s13326-017-0120-6)
Supplement: Additional file 1: — Table A. Sample tweets annotated in the gold standard for each sentiment category (DOCX 43 kb) [file 13326_2017_120_MOESM1_ESM.docx]

**Table A** Sample tweets annotated in the gold standard for each sentiment category

| **Sentiment** | | **Sample Tweets** |
| --- | --- | --- |
| Positive | | “Gardasil/HPV Vaccine SAFE: Most comprehensive Study To Date”  “Save lives by getting children the HPV vaccine” |
| Negative | Safety | “Study reveals 'unaoindable' danger of HPV vaccines”  “This mother wants you to see what an HPV vaccine injury looks like” |
|  | Efficacy | “Flat outlie that hpv vax is anti cancer vaccine. It has never been proven to prevent cancer. Never.”  “ACOG is now "recommending" ob/gyn's to push HPV vaccine despite it's ineffectivness &amp; it's notorious track record of killing &amp; maiming ppl” |
|  | NegCost | “Apparently my insurance would rather pay for my potential cervical cancer treatment than pay for my HPV vaccine. Tight.”  “Why 3 doses? Too troublesome” |
|  | Others | “I slap the hpv vaccine out of the nurses hand didn't you read the cable guys tweet you fool”  “Lead Developer of HPV Vaccines Comes Clean, Warns Parents &amp; Young Girls It’s All A Giant Deadly Scam” |
| Neutral | | “About I of 10 NJ boys received all 3 doses of HPV vaccine”  “Anyone know why Brazil isn’t vaccinating boys against HPV too?” |
| Unrelated | | “I am very healthy and HPV and HIV is negative (duh) lol”  “Approximately 80% of sexually active individuals will eventually contract at least 1 of the 100 strains of HPV” |
